# Phase II study of nedaplatin and amrubicin as first‐line treatment for advanced squamous cell lung cancer

**DOI:** 10.1111/1759-7714.13134

**Published:** 2019-07-16

**Authors:** Hirokazu Taniguchi, Hiroyuki Yamaguchi, Yosuke Dotsu, Midori Shimada, Hiroshi Gyotoku, Hiroaki Senju, Shinnosuke Takemoto, Takeshi Kitazaki, Masaaki Fukuda, Daiki Ogawara, Hiroshi Soda, Katsumi Nakatomi, Nanae Sugasaki, Akitoshi Kinoshita, Seiji Nagashima, Takaya Ikeda, Yoichi Nakamura, Noriho Sakamoto, Yasushi Obase, Minoru Fukuda, Hiroshi Mukae

**Affiliations:** ^1^ Department of Respiratory Medicine Nagasaki University Graduate School of Biomedical Sciences Nagasaki Japan; ^2^ Department of Respiratory Medicine The Japanese Red Cross Nagasaki Genbaku Hospital Nagasaki Japan; ^3^ Department of Respiratory Medicine Sasebo City General Hospital Sasebo Japan; ^4^ Department of Respiratory Medicine Ureshino Medical Center Ureshino Japan; ^5^ Department of Respiratory Medicine Nagasaki Prefecture Shimabara Hospital Shimabara Japan; ^6^ Department of Respiratory Medicine National Hospital Organization Nagasaki Medical Center Nagasaki Japan; ^7^ Department of Thoracic Oncology National Cancer Center Hospital East Kashiwa Japan; ^8^ Division of Thoracic Oncology Tochigi Cancer Center Utsunomiya Japan; ^9^ Clinical Oncology Center Nagasaki University Hospital Nagasaki Japan

**Keywords:** Amrubicin, clinical trial, nedaplatin, squamous cell lung cancer

## Abstract

**Background:**

The first‐line treatment for squamous cell lung cancer (SCC) has not necessarily been established; however, our previous exploratory study suggested that the combination of nedaplatin and amrubicin would be a promising treatment approach for patients with SCC. Therefore, a phase II study of this chemotherapeutic combination was designed to evaluate its efficacy and safety for treatment‐naïve patients with advanced SCC.

**Methods:**

A total of 21 treatment‐naïve patients with stage IIIB/IV or postoperative recurrent SCC were enrolled from six institutions. Nedaplatin (100 mg/m^2^) on day 1 and amrubicin (25 mg/m^2^) on days 1–3 were administered intravenously every 4 weeks. The primary endpoint was overall response rate (ORR), while the secondary endpoints included overall survival (OS), progression‐free survival (PFS), and drug toxicities.

**Results:**

Partial response was observed in seven of 21 cases (ORR, 33.3%; 95% confidence interval [CI], 14.5–52.2). Disease control rate, which includes stable disease, was 71.4%. Median OS and PFS was 14.6 and 4.1 months, respectively. This regimen did not cause any treatment‐related deaths. Grade 3/4 neutropenia developed in 8 of 21 cases (38.1%); however, febrile neutropenia developed in only 9.5% of the cases. Grade 3/4 gastrointestinal or neuromuscular toxicities were not observed.

**Conclusion:**

The efficacy of the combination of nedaplatin and amrubicin was comparable to that of other conventional chemotherapeutic regimens for treatment‐naïve patients with advanced SCC, and no severe gastrointestinal or neuromuscular toxicities were observed. This combination therapy may be an alternative treatment approach, particularly in patients who cannot tolerate gastrointestinal or neuromuscular toxicities.

## Introduction

Squamous cell lung cancer (SCC) accounts for approximately 20%–30% of all lung cancers. However, in contrast to the notable advances in the treatment of metastatic non‐SCC due to genetic alterations, the first‐line treatment of SCC has not necessarily been established. Although the efficacy of immune checkpoint inhibitors has been previously demonstrated, platinum‐based cytotoxic therapy is still considered a cornerstone of the treatment of SCC.^1^ In clinical practice, the conventional regimens for SCC include a combination of cisplatin or carboplatin with taxanes, gemcitabine, and S‐1 based chemotherapy. However, the median overall survival remains approximately 12–17 months, regardless of the occurrence of severe adverse events.^2^, ^3^, ^4^, ^5^, ^6^, ^7^ Therefore, a novel combination of anti‐cancer agents leading to fewer severe adverse events is required for the treatment of SCC patients.

Nedaplatin, a second‐generation platinum compound, has been developed to decrease the nephrotoxicity and gastrointestinal toxic effects induced by cisplatin, without affecting its anti‐tumor property.^8^, ^9^, ^10^ A randomized phase III study involving SCC patients showed that overall survival was longer with a combination of nedaplatin and docetaxel than with a combination of cisplatin and docetaxel.^11^ Amrubicin is a novel DNA topoisomerase II inhibitor; our previous phase I/II study revealed that a chemotherapeutic combination of amrubicin and nedaplatin was well tolerated, and that the overall response rate (ORR) was 48.6% (17 of 35 cases) for advanced non‐small cell lung cancer.^12^ The subset analysis (unpublished data) also suggested that the combination of nedaplatin and amrubicin was more effective in treating SCC (ORR, 70.0%; 7 of 10 cases) compared to non‐SCC (ORR, 40.0%; 10 of 25 cases). However, the number of SCC patients in that study was too small to evaluate the efficacy of nedaplatin and amrubicin for SCC. Thus, the present study was designed to clarify the efficacy and safety of a combination therapy with nedaplatin and amrubicin for SCC.

## Methods

### Patients and eligibility criteria

The eligibility criteria for this study were as follows: histologically confirmed stage IIIB/IV or postoperative recurrent SCC not amenable to curative radiotherapy (according to the 7th edition of the General Rule for Clinical and Pathological Record of Lung Cancer); no prior chemotherapy or recurrence more than 6 months after a previous adjuvant chemotherapy; no radiation therapy for a primary tumor; Eastern Cooperative Oncology Group (ECOG) performance status (PS) of 0 or 1; having measurable lesions according to the response evaluation criteria in solid tumors (RECIST) 1.1; age between 20 and 75 years; life expectancy of 12 weeks or more; adequate major organ function; white blood cell count ≥4 × 10^3^ cells/μL; absolute neutrophil count ≥2 × 10^3^ cells/μL; hemoglobin concentrations ≥9 g/dL; platelet count ≥100 × 10^3^ cells/μL; total bilirubin concentration ≤1.5 mg/dL; aspartate aminotransferase and alanine aminotransferase concentration ≥2 times the upper limit of the normal range; creatinine concentration ≤ the upper limit of the normal range; and arterial oxygen saturation at room air using pulse oxymetry ≥ 95%.

The major exclusion criteria were as follows: pulmonary fibrosis with an obvious shadow on chest radiography; uncontrollable fever; serious comorbidities including uncontrollable hypertension, diabetes mellitus or cardiovascular disease, active infection, mental disorder; uncontrollable pericardial effusion, pleural effusion, or ascites; second malignancy; pregnancy; lactation; history or presence of hemoptysis or bloody sputum; tumor invading or abutting major blood vessels; history of radiation therapy for lung field; or coexistence or history of interstitial lung diseases.

This study was conducted in accordance with the Declaration of Helsinki. The study protocol was reviewed and approved by the institutional review boards of the participating institutions, and a written informed consent was obtained from all patients. This trial was registered with University Hospital Medical Information Network (UMIN000003282).

### Treatment and assessment

All patients received nedaplatin (100 mg/m^2^) intravenously on day 1 and amrubicin (25 mg/m^2^) on days 1–3 every 4 weeks. Each patient received a minimum of three cycles until the onset of a progressive disease or unacceptable toxicity. The maximal number of chemotherapy cycles was six; however, the patients underwent more cycles, if necessary.

Before treatment, all patients underwent a complete medical history and physical examination, chest radiography, chest and abdominal computed tomography (CT), a radionuclide bone scan or positron emission tomography CT, brain CT or magnetic resonance imaging (MRI), and electrocardiography. Complete blood cell counts and blood chemistry studies were also conducted and repeated at least twice a week until treatment discontinuation. Scans or radiographs were obtained every 4–6 weeks to assess the overall response.

The response was investigator‐determined according to RECIST version 1.1. All adverse events were recorded and classified by grade according to the Common Terminology Criteria for Adverse Events (CTCAE) version 4.0.

### Statistical analysis

The primary endpoint was ORR. A Simon optimal two‐stage design was chosen for the determination of the total number of patients required for this study. Assuming an ORR of 20% for standard therapy, a target response rate of 40% was established. With alpha = 0.05 and beta = 0.20, the estimated number of patients required was 33. Considering unfitness, drop‐out, and discontinuation, the sample size of this study was determined to be 35. The secondary endpoints included overall survival (OS), progression‐free survival (PFS), and toxicities. OS was defined as the time from treatment start to death from any cause. PFS was defined as the time from treatment start to either progressive disease or death, whichever came first. All patients were followed‐up to May 2018, with a median follow‐up time being 14.5 months (95% confidence interval [CI], 6.2–27.9 months). The survival curves were plotted using the Kaplan‐Meier method with GraphPad Prism 7 (GraphPad Software Inc., La Jolla, CA, USA).

## Results

### Patient characteristics

A total of 21 patients from six institutions were enrolled between January 2012 and March 2016 (Table 1). The study population mainly comprised men with a current or previous history of smoking. The median age was 66 years, ranging from 55 to 75 years.

**Table 1:** Patient characteristics

	Number of patients (*n* = 21)	%
Age		
Median	66	
Range	55–75	
Sex		
Men	19	90.5
Women	2	9.5
ECOG PS		
0	6	28.6
1	15	71.4
Stage		
III	8	38.1
IV	12	57.1
Recurrence	1	4.8
Smoking status		
Current or former	21	100.0
Never	0	0.0

ECOG, Eastern Cooperative Oncology Group; PS, performance status.

### Response and survival

**Figure 1:**
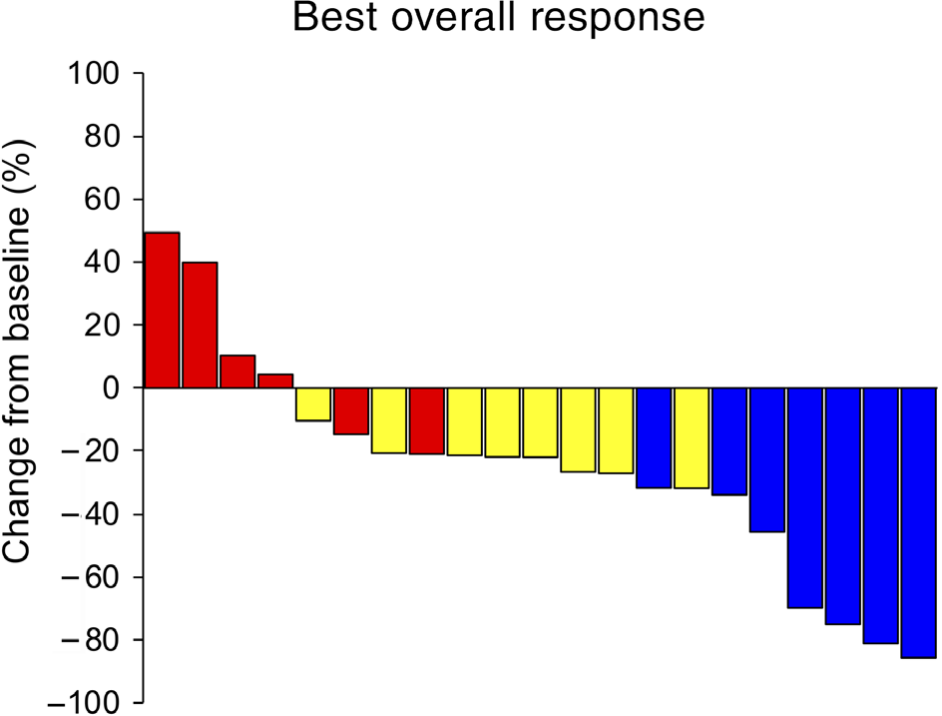
Waterfall plot of most favorable tumor changes from baseline. Each bar represents a patient. 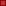 PD, progressive disease; 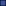 PR, partial response; 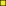 SD, stable disease.

**Figure 2:**
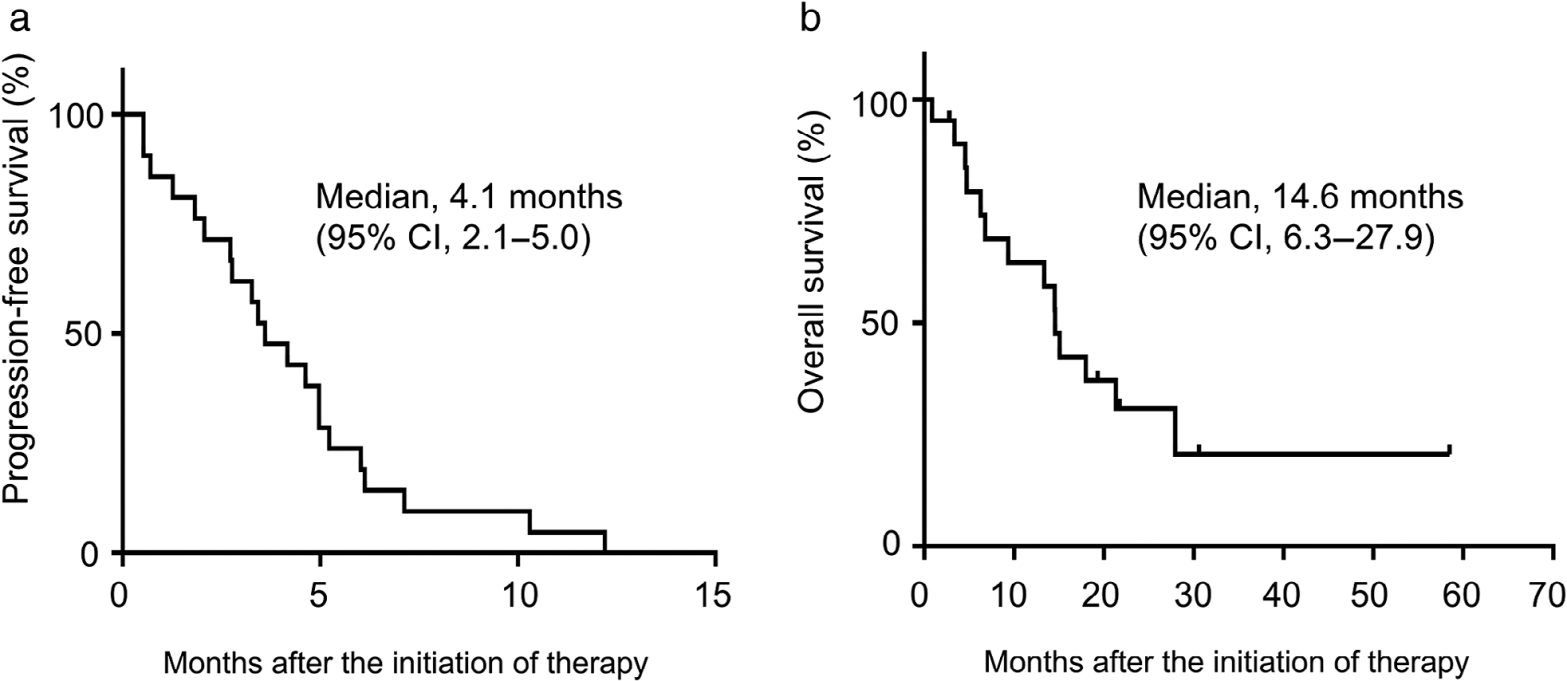
**(a)** Kaplan–Meier estimates of progression‐free survival. **(b)** Kaplan–Meier estimates of overall survival. The vertical bars indicate censored cases. CI, confidence interval

No patient showed a complete response, and seven patients showed partial responses (Table 2). The ORR was 33.3% (95% CI, 14.5–52.2%). Disease control rate, which includes stable disease, was 71.4% (95% CI, 53.1–89.8%). Figure 1 shows a waterfall plot of the most favorable tumor changes from baseline. According to the Kaplan‐Meier method, the median PFS was 4.1 months (95% CI, 2.1–5.0 months; Fig 2a) and the median OS was 14.6 months (95% CI, 6.3–27.9 months; Fig 2b). The median number of courses given per patient was four (range, 1–6). The second line therapy comprised docetaxel in nine cases, S‐1 based chemotherapy in four cases, nivolumab in two cases, nab‐paclitaxel in one case and local therapy to pleural effusion in the other.

**Table 2:** Response to the treatment

Best response	Number of patients	% (95% CI)
Complete response	0	0 (0–10.5)
Partial response	7	33.3 (14.5–52.2)
Stable disease	8	38.1 (18.8–57.4)
Progressive disease	6	28.6 (10.2–46.9)

CI, confidence interval.

### Adverse events

Hematological toxicities were more frequent than nonhematological toxicities (Table 3). Neutropenia appeared as a major severe hematological adverse event in eight of 21 cases (38.1%), although febrile neutropenia occurred only in 9.5%. The other grade 3/4 hematological toxicities included thrombocytopenia and anemia (9.5% each). They were successfully managed via supportive therapies. We also observed grade 3/4 nonhematological toxicities comprising hypernatremia and pneumonitis (4.8% each). Severe gastrointestinal and neuromuscular toxicities were not observed, and treatment‐related deaths did not occur.

**Table 3:** Toxicities occurring in all treatment courses

	Number of patients (%)	
Toxicities	Grade 1/2	Grade 3/4
Hematological adverse events		
Neutropenia	2 (9.5)	8 (38.1)
Thrombocytopenia	5 (23.8)	2 (9.5)
Anemia	3 (14.3)	2 (9.5)
Febrile neutropenia	1 (4.8)	2 (9.5)
Nonhematological adverse events		
Nausea	6 (28.6)	0 (0.0)
Fever	3 (14.3)	0 (0.0)
Constipation	3 (14.3)	0 (0.0)
Hiccups	3 (14.3)	0 (0.0)
Malaise	3 (14.3)	0 (0.0)
Hypernatremia	0 (0.0)	1 (4.8)
Hyponatremia	3 (14.3)	0 (0.0)
Pneumonitis	0 (0.0)	1 (4.8)
Nervous system disorders	0 (0.0)	0 (0.0)
Arthralgia	0 (0.0)	0 (0.0)
AST increased	1 (4.8)	0 (0.0)
ALT increased	1 (4.8)	0 (0.0)
ALP increased	1 (4.8)	0 (0.0)
γ‐GTP increased	3 (14.3)	0 (0.0)
LDH increased	1 (4.8)	0 (0.0)
Stomach pain	1 (4.8)	0 (0.0)
GERD	1 (4.8)	0 (0.0)
Vasculitis	1 (4.8)	0 (0.0)
Oral pain	1 (4.8)	0 (0.0)

ALP, alkaline phosphatase; ALT, alanine aminotransferase; AST, aspartate aminotransferase; GERD, gastroesophageal reflux disease; γ‐GTP, γ‐glutamyl transpeptidase; LDH, lactate dehydrogenase.

## Discussion

This study revealed the following important findings. The efficacy of the combination therapy including nedaplatin and amrubicin was comparable to that of other conventional chemotherapeutic regimens for treatment‐naïve patients with advanced SCC. The adverse events of this combination therapy were relatively few, with severe gastrointestinal and neuromuscular toxicities not being observed.

The current phase II study showed an ORR of 33.3% and 14.6 months of median OS. The subset analysis in SCC patients from a phase III study showed an ORR of 27.3% and median OS of 14.0 months in the carboplatin and S‐1 group, compared with an ORR of 33.9% and median OS of 10.6 months in the carboplatin and paclitaxel group.^13^ Subset analysis in SCC patients from another phase III study indicated that an ORR of 41% and median OS of 10.7 months were achieved in the carboplatin and *nab*‐paclitaxel group, whereas an ORR of 24% and median OS of 9.5 months were noted in the carboplatin plus paclitaxel group.^6^

Contrary to cisplatin‐ or carboplatin‐based regimens, the combination of nedaplatin and amrubicin could potentially benefit SCC patients. First, a meta‐analysis on nedaplatin‐based regimens revealed that ORR was 55.6% (95% CI, 52.5–58.7%) in SCC and 34.4% (95% CI, 32.3–36.5%) in non‐SCC, suggesting a higher efficacy of nedaplatin in SCC patients.^14^ Second, SCC patients could receive docetaxel or S‐1 as a second‐line treatment, if either is not employed as first‐line treatment. Docetaxel or S‐1 is considered effective in patients with refractory non‐small cell lung cancer after platinum‐based chemotherapy.^15^, ^16^, ^17^ Last, nedaplatin and amrubicin combination therapy caused lesser gastrointestinal or neuromuscular toxicities than did cisplatin‐ or taxane‐based regimens.^5^, ^11^

The current standard first‐line treatment for advanced SCC includes the combination of immune‐checkpoint inhibitors: pembrolizumab with carboplatin along with either paclitaxel or nab‐paclitaxel.^18^ However, all patients cannot undergo the above treatment due to adverse events or the presence of underlying diseases. Nedaplatin and amrubicin together would be useful in those unable to tolerate gastrointestinal or neuromuscular toxicities.

The present study has some limitations. First, we had to terminate the study prematurely due to the expected introduction of immune checkpoint inhibitors in clinical practice or clinical trials, because of which we failed to achieve the sample size of this study. The main reason was that the patients declined the proposal for this clinical trial. Therefore, the present findings are potentially subject to selection bias, and should be interpreted with caution. Second, the late line therapy of immune‐checkpoint inhibitors may affect overall survival time; however, only two patients received immune‐checkpoint inhibitors following this trial.

In conclusion, this is the first prospective study to demonstrate the clinical impact of a combination therapy of nedaplatin and amrubicin in SCC patients. Its efficacy was comparable to that of the other conventional regimens for SCC, and no severe gastrointestinal or neuromuscular toxicities were observed. These findings may pave the way for future treatment using the combination of nedaplatin and amrubicin with a novel anti‐cancer agent in some patients with SCC.

## Disclosure

The authors have no conflicts of interest to declare for this study.
